# School Outrages

**Published:** 1875-11

**Authors:** 


### SCHOOL OUTRAGES.

Thirty years ago a schoolmistress, in a rage, caught hold of the arm of a little girl not in fault, gave it a violent jerk, and, with a swing, threw her to the other side of the room. To-day, that little girl is a wife, a mother, the accomplished mistress of a princely mansion, happy in her social position, happy in her husband—who is one of the best of men—but that arm hangs powerless at her side, as it has done from the days of her childhood.

Two years ago, a beautiful young girl, just budding into womanhood, was going to school in mid-winter; she, with other scholars, was sent out for recreation for half an hour, as was the daily custom. Not knowing any better, she sat on a stone step in the sun, and daily did so. Thus, coming from a warm schoolroom, and remaining still in the open air until most thoroughly chilled, she acquired a permanent cough. She sleeps in the churchyard now. How many bright hopes have been blasted, how many an only child has been sent to an early grave, by ignorant, careless, and incompetent teachers, is frightful to think of.

